# Obituary

**Published:** 1904-08-15

**Authors:** 


### OBITUARY.

Dr. Henry C. Howells died in Hamilton, Ohio, June 23rd, 1904. Dr. Howells was one of the oldest practitioners in the State. He began practice in 1838, and settled in Hamilton in 1840, where he continued actively in practice up to the date of his death. He was almost 88 years of of age, and worked in his office the day before he passed away. He was not widely known in the profession, but was a faithful servant of the people of the community where he lived. He had many warm personal friends, who have wondered at the tenacious hold he had on life, and the ability to follow his professional work to the very close of a long and useful career. His must have been a strong constitution to withstand the exacting strain of so long a career in our profession; most of us will wear out in much less time. He accomplished his work in a quiet and unobtrusive manner, but yet not less effectively than others who have been more conspicuous.

Dr. I. P. Wilson.—Dr. Wilson was one of the prominent members of the profession in the West, and died at his home in Burlington, Iowa, March 9th, 1904. He was a teacher in Iowa University for many years, associated with Dr. A. O. Hunt and the late Dr. Kulp. He was a pleasant man socially and an earnest worker and teacher. He loved the profeession and gave his best thought to it. He was a frequent attendant of the national meetings, and will be missed from his local and State societies.

